# Mechanisms of Apoptosis-Related Long Non-coding RNAs in Ovarian Cancer

**DOI:** 10.3389/fcell.2021.641963

**Published:** 2021-04-29

**Authors:** Toshihiko Takeiwa, Kazuhiro Ikeda, Kuniko Horie-Inoue, Satoshi Inoue

**Affiliations:** ^1^Division of Systems Medicine & Gene Therapy, Saitama Medical University, Saitama, Japan; ^2^Department of Systems Aging Science and Medicine, Tokyo Metropolitan Institute of Gerontology, Tokyo, Japan

**Keywords:** ovarian cancer, ovary, apoptosis, long non-coding RNA, transcriptional regulation, histone modification complex, competing endogenous RNA

## Abstract

Ovarian cancer is a health-threatening malignancy of ovary in female reproductive systems and one of the most common gynecological malignancies worldwide. Due to rare early symptoms, ovarian cancers are often diagnosed at advanced stages and exhibit poor prognosis. Thus, efforts have been paid to develop alternative diagnostic and therapeutic strategies for the disease. Recent studies have presented that some long non-coding RNAs (lncRNAs) play roles in apoptosis of ovarian cancer cells through various mechanisms involved in the regulation of transcription factors, histone modification complexes, miRNAs, and protein stability. Because evasion of apoptosis in cancer cells facilitates to promote tumor progression and therapy resistance, apoptosis regulatory mechanisms of lncRNAs may be promising new targets in ovarian cancer. In this review, we introduce the recent findings in regard to the molecular mechanisms of apoptosis-related lncRNAs in ovarian cancer cells.

## Ovary and Ovarian Cancer

The ovary is a female reproductive organ where oocyte development occurs (Motta et al., 1997; Virant-Klun, 2015; Yadav et al., 2018) and functions as an endocrine organ involved in the synthesis of the female sex steroid hormones and the regulation of reproduction such as the menstrual cycle, pregnancy, and lactation (Hiller-Sturmhöfel and Bartke, 1998). Thus, the health of ovaries is essential for reproduction and women’s lives, rendering finding cures to ovarian diseases crucial. Ovarian cancer is one of the most common gynecological cancers (Momenimovahed et al., 2019). The GLOBOCAN 2018 data estimates ∼300,000 new cases of ovarian cancer and over 180,000 ovarian cancer–related deaths per year worldwide (Bray et al., 2018). Ovarian cancer is a heterogeneous disease and classified by type of originated cell. Epithelial ovarian cancer (EOC) is the most common ovarian cancer (∼90%) (Rojas et al., 2016; Momenimovahed et al., 2019). The disease is often advanced at diagnosis due to lack of early symptoms and the 5-year cause-specific survival rate is <50% (Siegel et al., 2018; Torre et al., 2018; Trinidad et al., 2020). Based on the current limitations, alternative diagnostic and therapeutic approaches for ovarian cancer remain to be explored.

## Apoptosis in Ovary and Ovarian Cancer

Apoptosis is a process of programmed cell death triggered by intrinsic or extrinsic signals (Wong, 2011). Intrinsic signals are initiated by cellular stresses. These signals increase the mitochondrial permeability and release of the pro-apoptotic factors such as cytochrome-c, resulting in activation of cysteine-aspartic acid proteases (caspases), which are essential enzymes for apoptosis execution. Meanwhile, extrinsic signals are mediated by death receptor signaling pathways. Death receptors, their ligands, and adaptor proteins form the death-inducing signaling complex (DISC), which triggers caspase activation (Wong, 2011).

Apoptosis plays physiological roles in normal ovary functions such as follicular atresia and corpus luteum regression (Vaskivuo and Tapanainen, 2003; Yadav et al., 2018). In malignant tumors, evasion of apoptosis facilitates cancer cell survival and tumor progression (Wong, 2011; Binju et al., 2019), thus efforts have been paid for cancer strategies to discover the molecules to exert apoptosis in cancer cells whereas not in normal cells. For ovarian cancer treatment, small chemicals that modulate apoptosis-related proteins such as inhibitors of apoptotic proteins (IAPs) have entered clinical trials (Binju et al., 2019).

In terms of apoptosis pathways in cancers, several mechanisms of apoptosis-related genes have been well characterized. Transcription factors such as E2F family proteins, nuclear factor kappa B (NF-κB) proteins, and signal transducer and activator of transcription (STAT) family proteins modulate apoptosis via regulating transcription of apoptosis-related genes (Bours et al., 2000; Crosby and Almasan, 2004; Karin, 2006; Kim and Lee, 2007; Kent and Leone, 2019; Verhoeven et al., 2020). Histone modification complexes such as polycomb repressive complex 1/2 (PRC1/2) affect transcription of apoptosis-related genes through histone methylation (Cao et al., 2011; Wang W. et al., 2015; Christofides et al., 2016). Apoptosis-related genes are also modulated by post-transcriptional gene regulation mechanism, such as through miRNAs that regulate apoptosis-related gene mRNAs (Di Leva et al., 2014; Pistritto et al., 2016; Si et al., 2019). Ubiquitin-mediated protein degradation systems are also involved in apoptosis (Zhang et al., 2004; Hoeller and Dikic, 2009; Yang et al., 2009), as some E3 ubiquitin ligases are involved in ubiquitination of apoptosis-related proteins (Hoeller and Dikic, 2009; Yang et al., 2009, 2018; Woo and Kwon, 2019).

Considering the importance of apoptosis in cancer pathophysiology, strategies targeting these apoptosis regulatory mechanisms may contribute to the development of novel ovarian cancer therapies.

## Long Non-Coding RNA (lncRNA)

**FIGURE 1:**
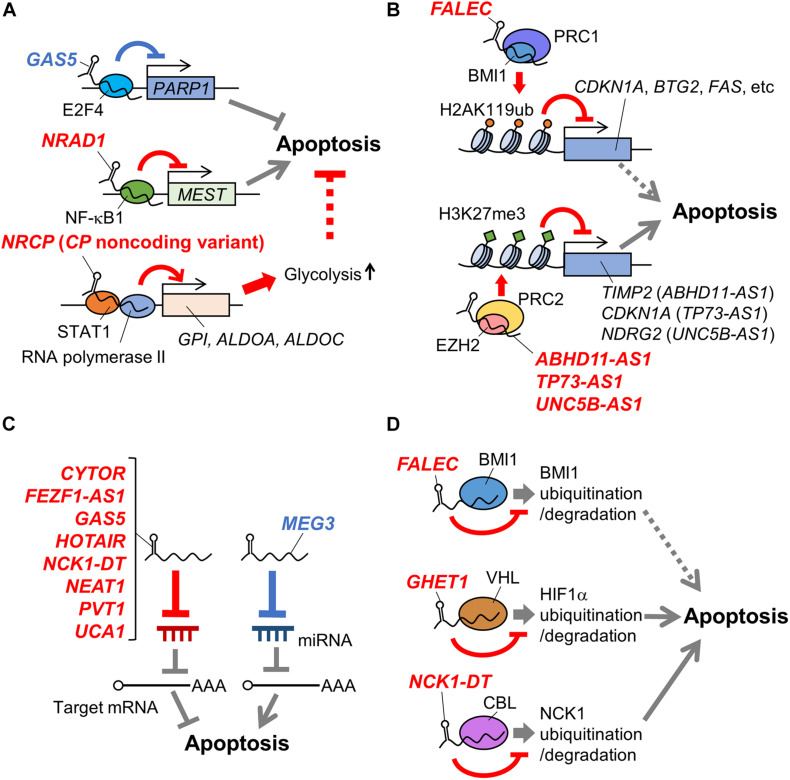
Schematic representation of mechanisms of apoptosis-related lncRNAs in ovarian cancer. LncRNAs involved in apoptosis of ovarian cancer cells via regulating transcription factors **(A)**, histone modification complexes **(B)**, miRNAs **(C)**, and protein stability **(D)** are shown. Names of lncRNAs promoting and suppressing apoptosis are shown in blue and red, respectively. In addition, apoptosis-promotive or suppressive functions of lncRNAs are shown in blue or red lines, respectively. Potential apoptosis-associated biological pathways are shown in dotted lines. *ABHD11-AS1, abhydrolase domain containing 11 antisense RNA 1; ALDOA, aldolase, fructose-bisphosphate A; ALDOC, aldolase, fructose-bisphosphate C*; BMI1, B lymphoma Mo-MLV insertion region 1 homolog; *BTG2, B-cell translocation gene 2*; CBL, casitas B-lineage lymphoma: *CDKN1A, cyclin dependent kinase inhibitor 1A; CP, ceruloplasmin; CYTOR, cytoskeleton regulator RNA*; EZH2, enhancer of zeste homolog 2; *FALEC, focally amplified long non-coding RNA in epithelial cancer; FEZF1-AS1, fasciculation and elongation protein zeta family zinc finger 1 antisense RNA 1; GAS5, growth arrest-specific 5; GHET1, gastric carcinoma high expressed transcript 1; GPI, glucose-6-phosphate isomerase*; H2AK119ub, the ubiquitination at the 119th lysine residue of the histone H2A; H3K27me3, the tri-methylation at the 27th lysine residue of the histone H3; HIF1α, hypoxia-inducible factor 1α; *HOTAIR, HOX transcript antisense RNA; MEG3, maternally expressed gene 3; MEST, mesoderm specific transcript*; NCK1, non-catalytic region of tyrosine kinase adaptor protein 1; *NCK1-DT, NCK1 divergent transcript; NDRG2, n-myc downstream-regulated gene 2; NEAT1, nuclear enriched abundant transcript 1*; NF-κB1, nuclear factor kappa B subunit 1; *NRAD1, non-coding RNA in the aldehyde dehydrogenase 1A pathway; NRCP, lncRNA ceruloplasmin; PARP1, poly(ADP-ribose) polymerase 1; PVT1, plasmacytoma variant translocation 1*; STAT1, signal transducer and activator of transcription 1; *TIMP2, tissue inhibitor of metalloproteinase 2; TP73-AS1, tumor protein p73 antisense RNA 1; UCA1, urothelial carcinoma associated 1; UNC5B-AS1, uncoordinated 5 netrin receptor B antisense RNA 1*.

Long non-coding RNAs (lncRNAs) are defined as >200-nt transcripts that do not encode proteins and tens of thousands of lncRNA transcripts are identified throughout the human genome, the majority with unknown function. However, functional studies of some lncRNAs have revealed that they have a wide range of functions. For example, lncRNAs regulate transcription and chromatin remodeling by modulating the recruitment of transcription factors and PRC to specific genomic loci. Furthermore, lncRNAs are involved in gene regulation at post-transcriptional levels through interacting with mRNAs, miRNAs, and proteins (Marchese et al., 2017). Intriguingly, lncRNAs play important roles in pathophysiology of various cancers (Takayama and Inoue, 2016; Misawa et al., 2017; Arun et al., 2018; Mitobe et al., 2018; Kamada et al., 2020; Takeiwa et al., 2020). Particularly, several lncRNAs have been suggested to regulate the apoptosis of ovarian cancer cells (Figure 1 and Table 1). In the following sections, we will describe some apoptosis-related lncRNAs in ovarian cancer cells according to their mechanisms.

**TABLE 1:** Mechanisms of apoptosis-related lncRNAs in ovarian cancer cells.

lncRNA	Chr. location	Roles in ovarian cancer cells/xenograft tumors	Clinical relevance in ovarian cancer tissues/patients	Effects on apoptosis
*GAS5*	1q25	↓ in A2780/HEY/HO8910 ^*a*^/OVCAR3/SKOV3 cells Binds to E2F4 and represses *PARP1* in HEY/SKOV3 cells Sponges *miR-196a-5p* to upregulate *HOXA5* in primary tumor cells from HGSOV tissues and A2780/OVCAR3 cells Represses PARP1, growth and cisplatin resistance in SKOV3 tumors	↓ in tumor tissues ↓ is correlated with shorter DFS/OS	+
*NRAD1/LINC00284*	13q14	↑ in A2780/CAOV3/HO8910/OVCAR3/SKOV3 cells Binds to NF-κB1 and represses *MEST* in HO8910 cells Promotes growth of HO8910 tumors	↑ in ovarian cancer tissues	−
*CP* non-coding variant *(NRCP)*	3q24-25	↑ in A2780/IGROV1/OVCAR3/SFMAR/SFWAS/SKOV3 cells Binds to RNA Pol II and STAT1, upregulates *GPI, ALDOA*, and *ALDOC* Promotes growth and metastasis of A2780/SKOV3 tumors	↑ in tumor tissues ↑ is correlated with shorter OS	−
*ABHD11-AS1*	7q11	↑ in HO8910/OVCA429 cells Upregulates RhoC in A2780/OVCAR3 cells Binds to EZH2 and represses *TIMP2* in HO8910/OVCA429 cells Promotes growth and metastasis of A2780 tumors	↑ in tumor tissues	−
*FALEC/FAL1*	1q21	Promotes PRC1-mediated repression of *CDKN1A, BTG2*, and *FAS* in A2780 cells Binds to and stabilizes BMI1 in A2780 cells Promotes A2780 tumor growth	↑ and copy number gain correlated with shorter OS	−
*TP73-AS1*	1p36	↑ in CAOV3/HO8910/OV420/SKOV3 cells Binds to EZH2 and represses *CDKN1A* in SKOV3 cells Promotes SKOV3 tumor growth	↑ in tumor tissues ↑ is correlated with poor prognosis	−
*UNC5B-AS1*	10q22	↑ in A2780/ES2/SKOV3 cells Binds to EZH2 and promotes *NDRG2* in ES2/SKOV3 cells	↑ in tumor tissues	−
*FEZF1-AS1*	7q31	↑ in A2780/COC1/PEO1/SKOV3 cells Sponges *miR-130a-5p*, upregulates *SOX4* in COC1/SKOV3 cells	↑ in tumors and serum ↑ is correlated with shorter OS	−
*HOTAIR*	12q13	↑ in cisplatin-resistant A2780/SKOV3 cells Sponges *miR-138-5p*, upregulates *EZH2* and *SIRT1* in A2780/SKOV3 cells Promotes A2780 tumor growth	↑ in tumor tissues ↑ is correlated with shorter OS in HGSOV patients	−
*CYTOR/LINC00152*	2p11	↑ in A2780/HO8910/SKOV3 cells Sponges *miR-125b* to upregulate *MCL1* in A2780/SKOV3 cells Increases MCL1 level and SKOV3 tumor growth	↑ in tumor tissues ↑ is correlated with shorter OS	−
*MEG3*	14q32	↓ in OVCAR3/OVCAR5/OVCAR8/SKOV3 cells Sponges *miR-205-5p* in OVCAR8/SKOV3 cells	↓ in tumor tissues	+
*NCK1-DT/NCK1-AS1*	3q22	↑ in CAOV3/OVCAR3/SKOV3/SNU119/SUN8 cells Sponges *miR-137* to upregulate *NCK1* in CAOV3/SKOV3 cells Prevents CBL-mediated NCK1 degradation in CAOV3/SKOV3 cells	↑ in tumor tissues	−
*NEAT1*	11q13	↑ in A2780/CAOV3/ES2/HO8910/OV90/OVCAR3/SKOV3 cells ↑ in paclitaxel-resistant HeyA8/SKOV3 cells versus parental cells ↑ in cisplatin-resistant A2780/SKOV3 cells versus parental cells Sponges *miR-34a-5p*, upregulates *BCL2* in OVCAR3/SKOV3 cells Sponges *miR-4500*, upregulates *BZW1* in CAOV3/ES2 cells Sponges *miR-194*, upregulates *ZEB1* in HeyA8/SKOV3 cells Sponges *miR-770-5p*, upregulates *PARP1* in A2780/SKOV3 cells Promotes growth of SKOV3/A2780 tumors and paclitaxel resistance	↑ in tumor tissues ↑ in cisplatin and paclitaxel-resistant cancer tissues ↑ is correlated with shorter OS	−
*PVT1*	8q24	↑ in A2780/OVCAR3/TOV112D cells Sponges *miR-543*, upregulates *SERPINI1* in OVCAR3/TOV112D cells	↑ in tumor tissues ↑ is correlated with shorter OS	−
*UCA1*	19p13	↑ in A2780/HeyA8/OAW42/OVCAR4/SKOV3 cells ↑ in paclitaxel-resistant HeyA8/SKOV3 cells ↑ in cisplatin-resistant A2780/SKOV3 cells Sponges *miR-129*, upregulates *ABCB1* in HeyA8/SKOV3 cells Sponges *miR-654-5p*, upregulates *SIK2* in HeyA8/SKOV3 cells Sponges *miR-143*, upregulates *FOSL2* in A2780/SKOV3 cells	↑ in tumor tissues ↑ in tumors and serum exosomes of patients with cisplatin-resistant cancers	−
*GHET1*	7q36	↑ in 3AO/A2780/OVCAR3/SKOV3 cells Prevents VHL-mediated HIF1α degradation in A2780/SKOV3 cells	↑ in tumor tissues ↑ is correlated with increased tumor size and distant metastasis	−

*^a^Although HO8910 cell line was used as an ovarian cancer cell model in these studies, a previous study has reported that it is a cross-contaminated cell line. ABCB1, ATP binding cassette subfamily B member 1; ABHD11-AS1, abhydrolase domain containing 11 antisense RNA 1; ALDOA, aldolase, fructose-bisphosphate A; ALDOC, aldolase, fructose-bisphosphate C; BCL2, B-cell lymphoma 2; BMI1, B lymphoma Mo-MLV insertion region 1 homolog; BTG2, B-cell translocation gene 2; BZW1, basic leucine zipper and W2 domain-containing protein 1; CBL, casitas B-lineage lymphoma; CDKN1A, cyclin dependent kinase inhibitor 1A; CP, ceruloplasmin; CYTOR, cytoskeleton regulator RNA; DFS, disease-free survival; EZH2, enhancer of zeste homolog 2; FAL1, focally amplified lncRNA on chromosome 1; FALEC, focally amplified long non-coding RNA in epithelial cancer; FEZF1-AS1, fasciculation and elongation protein zeta family zinc finger 1 antisense RNA 1; FOSL2, Fos-related antigen 2; GAS5, growth arrest-specific 5; GHET1, gastric carcinoma high expressed transcript 1; GPI, glucose-6-phosphate isomerase; HGSOV, high-grade serous ovarian cancer; HIF1α, hypoxia-inducible factor 1α; HOTAIR, HOX transcript antisense RNA; HOXA5, homeobox A5; LINC00152, long intergenic non-coding RNA 00152; LINC00284, long intergenic non-coding RNA 00284; MCL1, myeloid cell leukemia 1; MEG3, maternally expressed gene 3; MEST, mesoderm specific transcript; NCK1, non-catalytic region of tyrosine kinase adaptor protein 1; NCK1-AS1, NCK1 antisense RNA 1; NCK1-DT, NCK1 divergent transcript; NDRG2, n-myc downstream-regulated gene 2; NEAT1, nuclear enriched abundant transcript 1; NRAD1, non-coding RNA in the aldehyde dehydrogenase 1A pathway; NRCP, lncRNA ceruloplasmin; OS, overall survival; PARP1, poly(ADP-ribose) polymerase 1; PTX, paclitaxel; PVT1, plasmacytoma variant translocation 1; RhoC, ras homolog family member C; SERPINI1, serpin family I member 1; SIK2, salt inducible kinase 2; SIRT1, sirtuin 1; SOX4, sex-determining region Y (SRY)-box transcription factor 4; TIMP2, tissue inhibitor of metalloproteinase 2; TP73-AS1, tumor protein p73 antisense RNA 1; UCA1, urothelial carcinoma associated 1; UNC5B-AS1, uncoordinated 5 netrin receptor B antisense RNA 1; VHL, von Hippel–Lindau tumor suppressor; ZEB1, zinc finger E-box binding homeobox 1.*

### Apoptosis-Related LncRNAs Regulating Transcription Factors

In this section, we will introduce an apoptosis-promotive lncRNA *growth arrest-specific 5 (GAS5)* and apoptosis-suppressive lncRNAs *non-coding RNA in the aldehyde dehydrogenase 1A pathway (NRAD1)/long intergenic non-coding RNA 00284 (LINC00284)* and a non-coding variant of *ceruloplasmin (CP) (lncRNA ceruloplasmin; NRCP)*.

#### GAS5

*Growth arrest-specific 5* is downregulated in ovarian cancer, with this low expression associated with shorter disease-free period and lower overall survival rate of ovarian cancer patients (Gao et al., 2015; Li et al., 2016; Zhao et al., 2018; Long et al., 2019). *GAS5* overexpression promotes apoptosis of ovarian cancer cells such as A2780, HEY, OVCAR3, and SKOV3, and increases the sensitivity of HEY and SKOV3 cells to the anticancer agent cisplatin (Gao et al., 2015; Li et al., 2016; Zhao et al., 2018; Long et al., 2019). A functional study has shown that *GAS5* recruits the E2F4 transcription factor to the *poly(ADP-ribose) polymerase 1 (*PARP1*)* promoter, repressing *PARP1* transcription in HEY and SKOV3 cells (Long et al., 2019; Figure 1A). *GAS5*-mediated *PARP1* repression might contribute to apoptosis by downregulating the mitogen-activated protein kinase (MAPK) pathway, but direct evidence will be required in the future study.

#### NRAD1/LINC00284

*Non-coding RNA in the aldehyde dehydrogenase 1A pathway/long intergenic non-coding RNA 00284* is highly expressed in ovarian cancer. *NRAD1* overexpression and knockdown experiments have shown that it suppresses the apoptosis of HO8910 and OVCAR3 cells. Functional analyses using HO8910 cells have suggested that *NRAD1* binds to NF-κB subunit 1 (NF-κB1) transcription factor and induces NF-κB1–mediated transcriptional repression of *mesoderm specific transcript (MEST)*, contributing to the suppression of apoptosis (Ruan and Zhao, 2019; Figure 1A). However, since a previous study has reported that HO8910 is a cross-contaminated cell line, this mechanism is needed to be verified using other ovarian cancer models (Ye et al., 2015).

#### CP Non-coding Variant (NRCP)

*NRCP* is a non-coding splice variant of the ceruloplasmin-coding gene that is upregulated in ovarian cancer (Rupaimoole et al., 2015). High *NRCP* expression levels correlate with shorter overall survival in patients with ovarian cancer, while *NRCP* knockdown induces apoptosis in A2780 and SKOV3 cells (Rupaimoole et al., 2015). *NRCP* binds to RNA polymerase II and STAT1 transcription factor, and promotes glycolysis in A2780 and SKOV3 cells by upregulating glycolysis pathway genes such as *glucose-6-phosphate isomerase (GPI), aldolase, fructose-bisphosphate A (ALDOA)*, and *aldolase, fructose-bisphosphate C (ALDOC)* via STAT1 (Rupaimoole et al., 2015; Figure 1A). These results suggest a possibility that *NRCP* may modulate apoptosis by regulating cancer metabolism. *NRCP* is not annotated in National Center for Biotechnology Information (NCBI) Reference Sequence (RefSeq) database (on Feb 3rd, 2021) and requires further characterization of sequences and expression profiles.

### Apoptosis-Related LncRNAs Regulating Histone Modification Complexes

In this section, we will describe the following apoptosis-suppressive lncRNAs: *abhydrolase domain containing 11 antisense RNA 1 (ABHD11-AS1), focally amplified long non-coding RNA in epithelial cancer (FALEC)/focally amplified lncRNA on chromosome 1 (FAL1), tumor protein p73 antisense RNA 1 (TP73-AS1)*, and *uncoordinated 5 netrin receptor B antisense RNA 1 (UNC5B-AS1)*.

#### ABHD11-AS1

*Abhydrolase domain containing 11 antisense RNA 1* is upregulated in ovarian cancer (Wu et al., 2017; Zeng et al., 2019). A functional study has shown that *ABHD11-AS1* modulates the expression of ras homolog family member C (RhoC) by an unknown mechanism, suppressing apoptosis in A2780 and OVCAR3 cells (Wu et al., 2017). Another functional study has shown that *ABHD11-AS1* binds to enhancer of zeste homolog 2 (EZH2), a component of PRC2. *ABHD11-AS1* facilitates tri-methylation at the 27th lysine residue of the histone H3 protein (H3K27me3) on the *tissue inhibitor of metalloproteinase 2 (TIMP2)* promoter, as mediated by PRC2, and likewise suppresses *TIMP2* expression in HO8910 cells and OVCA429 ovarian cancer cells (Figure 1B). *TIMP2* suppression mediated by *ABHD11-AS1* promotes the proliferation of OVCA429 cells, suggesting that *ABHD11-AS1* may also modulate apoptosis by this mechanism (Zeng et al., 2019).

#### FALEC/FAL1

*Focally amplified lncRNA in epithelial cancer/focally amplified lncRNA on chromosome 1* was initially identified as an lncRNA whose gene copy number increased in multiple types of cancers, including ovarian cancer (Hu et al., 2014). Its high expression level and gain in genomic copy number correlate with a shorter overall survival rate of late-stage ovarian cancer patients (Hu et al., 2014). A functional study using A2780 cells has suggested that *FALEC* binds to a component of PRC1, B lymphoma Mo-MLV insertion region 1 homolog (BMI1) protein, and recruits PRC1 to the promoters of genes such as *cyclin dependent kinase inhibitor 1A (CDKN1A), B-cell translocation gene 2 (BTG2)*, and *FAS*. Subsequently, PRC1 mediates the ubiquitination at the 119th lysine residue of the histone H2A (H2AK119ub) on these promoter regions and the suppression of these genes (Figure 1B). The *FALEC*/PRC1 complex target genes such as *CDKN1A, BTG2*, and *FAS* are suggested to be involved in apoptosis regulation (El-Deiry, 2001; Mao et al., 2015). Thus, *FALEC* can be a regulator of ovarian cancer apoptosis.

#### TP73-AS1

*Tumor protein p73 antisense RNA 1* is upregulated in EOC and associated with poor prognosis in EOC patients (Li Y. et al., 2019). A recent study has shown that *TP73-AS1* knockdown induces apoptosis of SKOV3 cells, suppressing the proliferation in *in vitro* culture and the xenograft tumor formation in athymic mice. In contrast, *TP73-AS1* overexpression suppresses apoptosis in CAOV3 ovarian cancer cells. Functional analyses have suggested that *TP73-AS1* epigenetically suppresses *CDKN1A* expression by recruiting PRC2 to its promoter (Figure 1B) and modulates apoptosis of SKOV3 cells through this mechanism (Li Y. et al., 2019).

#### UNC5B-AS1

*Uncoordinated 5 netrin receptor B antisense RNA 1* is highly expressed in ovarian cancer, and a recent study has shown that its knockdown activates caspase 3 in ES2 and SKOV3 cells, suggesting the apoptosis-suppressive role of *UNC5B-AS1* (Wang et al., 2020). Moreover, the same study has suggested that *UNC5B-AS1* promotes PRC2 to repress the *n-myc downstream-regulated gene 2 (NDRG2)* expression epigenetically (Figure 1B), which may suppress ovarian cancer cell apoptosis (Wang et al., 2020). This study is limited in the elucidation of the mechanism by which *UNC5B-AS1* regulates PRC2 and its *in vivo* function, and further functional analyses are required.

### Apoptosis-Related LncRNAs Regulating miRNAs

In the section, we will introduce an apoptosis-promotive lncRNA GAS5 and the following apoptosis-suppressive lncRNAs: *fasciculation and elongation protein zeta family zinc finger 1 antisense RNA 1 (FEZF1-AS1), HOX transcript antisense RNA (HOTAIR), non-catalytic region of tyrosine kinase adaptor protein 1 (NCK1) divergent transcript (NCK1-DT)/NCK1 antisense RNA 1 (NCK1-AS1), nuclear enriched abundant transcript 1 (NEAT1)*, and *urothelial carcinoma associated 1 (UCA1)*.

#### FEZF1-AS1

High levels of *FEZF1-AS1* are detected in tumor tissues and the serum of EOC patients, with its high expression associated with shorter overall survival of EOC patients (Sun et al., 2020). Moreover, its knockdown promotes apoptosis in COC1 and SKOV3 ovarian cancer cells, suggesting the apoptosis-suppressive role of *FEZF1-AS1*. *In vitro* analyses of *FEZF1-AS1* have shown that it functions as a competing endogenous RNA (ceRNA) for *miR-130a-5p*, or sponges *miR-130a-5p* (Figure 1C). Consequently, *FEZF1-AS1* upregulates the expression of a *miR-130a-5p* target gene, *sex-determining region Y (SRY)-box transcription factor 4 (SOX4)*, that promotes proliferation of COC1 and SKOV3 cells and may contribute to apoptosis suppression (Sun et al., 2020). Further analysis of *FEZF1-AS1* function, especially *in vivo*, will clarify its role and significance in apoptosis of ovarian cancer cells.

#### GAS5

A recent functional study has suggested that *GAS5* functions as a ceRNA for *miR-196a-5p* to upregulate *homeobox A5 (HOXA5)*, promoting apoptosis of primary tumor cells from high-grade serous ovarian cancer tissues as well as A2780 and OVCAR3 cells (Zhao et al., 2018; Figure 1C).

#### HOTAIR

*HOX transcript antisense RNA* is upregulated in ovarian cancer, and the elevated expression level correlates with the shorter overall survival of ovarian cancer patients (Qiu et al., 2015; Wang Y. et al., 2015; Zhang et al., 2016; Luo et al., 2017; Yu et al., 2018). *HOTAIR* knockdown induces apoptosis in ovarian cancer cells such as A2780, HeyC2, and OVCA429, and decreases the cisplatin sensitivity of A2780 and SKOV3 cells (Qiu et al., 2015; Wang Y. et al., 2015; Zhang et al., 2016, 2020; Yu et al., 2018). A recent functional study using A2780 and SKOV3 cells has suggested that *HOTAIR* acts as a ceRNA for *miR-138-5p*, leading to cisplatin resistance of these cells (Zhang et al., 2020; Figure 1C). This study has shown that *HOTAIR/miR-138-5p* axis modulates *EZH2* and *sirtuin 1 (SIRT1)* expression, but its biological significance has not been elucidated.

#### NCK1-DT/NCK1-AS1

*Non-catalytic region of tyrosine kinase adaptor protein 1 divergent transcript* is highly expressed in ovarian cancer. Mechanistically, it acts as a ceRNA for *miR-137* to upregulate *NCK1*, which suppresses apoptosis of CAOV3 and SKOV3 cells and enhances their cisplatin resistance (Chang et al., 2020; Figure 1C).

#### NEAT1

*Nuclear enriched abundant transcript 1* is upregulated in ovarian cancer and is associated with shorter overall survival of ovarian cancer patients (Chen et al., 2016). *NEAT1* acts as a ceRNA for *miR-34a-5p* to upregulate BCL2 and suppresses apoptosis of OVCAR3 and SKOV3 cells (Ding et al., 2017). In addition, NEAT1 acts as a ceRNA for *miR-4500*, to upregulate *basic leucine zipper and W2 domain-containing protein 1 (BZW1)* that suppresses apoptosis of CAOV3 and ES2 cells (Xu et al., 2020), and *miR-194* to upregulate the transcription factor zinc finger E-box binding homeobox 1 (ZEB1), promoting resistance to the anticancer agent paclitaxel (PTX) in HeyA8 and SKOV3 cells (An et al., 2017). Furthermore, *NEAT1* sponges *miR-770-5p*, to upregulate *PARP1* and increase cisplatin resistance in A2780 cells *in vivo* (Zhu et al., 2020; Figure 1C).

#### UCA1

The lncRNA *UCA1* is upregulated in ovarian cancer and is detected in exosomes derived from the serum of ovarian cancer patients (Li Z. et al., 2019; Li et al., 2020). Functional studies have shown that UCA1 acts as a ceRNA for *miR-129* and *miR-654-5p* to upregulate *ATP binding cassette subfamily B member 1 (ABCB1)* and *SALT INDUCIBLE KINASE 2 (SIK2)*, respectively, which contribute to the suppression of apoptosis and the enhancement of PTX resistance in HeyA8 and SKOV3 cells (Wang et al., 2018; Li et al., 2020). In addition, *UCA1* functions as a ceRNA for *miR-143* to increase *Fos-related antigen 2 (FOSL2)*, and enhances cisplatin resistance in A2780 and SKOV3 cells (Li Z. et al., 2019; Figure 1C). However, the importance of the function of *UCA1* as a ceRNA *in vivo* has not been fully analyzed.

Recent studies have found that many other lncRNAs modulate ovarian cancer apoptosis through regulating miRNAs. For example, *CYTOR/LINC00152* acts as a ceRNA of *miR-125b* to upregulate an antiapoptotic protein MCL1 in A2780 and SKOV3 cells (Chen et al., 2018). *PVT1* suppresses apoptosis in OVACAR3 and TOV112D cells by inhibiting *miR-543* and increasing a *miR-543* target *SERPIN1* (Qu et al., 2020). In contrast, *MEG3* promotes apoptosis in OVCAR8 and SKOV3 cells by sponging *miR-205-5p* (Tao et al., 2020). The detail of lncRNAs regulating miRNAs is also reviewed in other articles (Braga et al., 2020; Salamini-Montemurri et al., 2020).

### Apoptosis-Related LncRNAs Regulating Protein Stability

In the section, we will introduce the following apoptosis-suppressive lncRNAs: *FALEC/FAL1*, *gastric carcinoma high expressed transcript 1 (GHET1)*, and *NCK1-DT/NCK1-AS1*.

#### FALEC/FAL1

As described above, *FALEC* binds to BMI1 and modulates PRC1 function in A2780 cells. In addition, *FALEC* stabilizes BMI1 by suppressing ubiquitin-mediated BMI1 protein degradation (Hu et al., 2014; Figure 1D).

#### GHET1

The lncRNA *GHET1* is upregulated in ovarian cancer and higher expression correlates with increased tumor size and distant metastasis (Liu and Li, 2019). Conversely, its knockdown induces apoptosis and downregulates glycolysis in A2780 and SKOV3 cells, where *GHET1* binds to an E3 ubiquitin ligase, von Hippel–Lindau tumor suppressor (VHL), and prevents VHL-mediated degradation of hypoxia-inducible factor 1α (HIF1α) (Figure 1D). Since the *GHET1* function in ovarian cancer cells has been only examined by *in vitro* assays, *in vivo* analyses of *GHET1* are needed. Although the role of the *GHET1*/VHL/HIF1α axis in apoptosis has not yet been elucidated, HIF1α and cancer metabolism have been shown to play important roles in apoptosis regulation, suggesting the possibility that this axis may also be involved in the phenomenon (Zhou et al., 2006; Matsuura et al., 2016).

#### NCK1-DT/NCK1-AS1

In addition to the function as a ceRNA, *NCK1-AS1* increases the stability of NCK1: *NCK1-AS1* binds to an E3 ubiquitin ligase, casitas B-lineage lymphoma (CBL), and prevents CBL-mediated degradation of NCK1 (Chang et al., 2020; Figure 1D). The functions of *NCK-AS1* in ovarian cancer have been suggested based on *in vitro* experiments, and thus needs to be evaluated using ovarian tumor specimens or *in vivo* ovarian cancer models.

## Conclusion

In this review, we introduced the mechanisms of apoptosis-related lncRNAs in ovarian cancer cells. Considering that dysregulation of apoptosis is involved in the resistance to ovarian cancer therapies, small molecule inhibitors/siRNAs targeting apoptosis-suppressing lncRNAs, or apoptosis-promoting lncRNAs themselves may be applicable to ovarian cancer therapies. For nucleic acid-based therapeutics, it is important to develop the drug delivery systems (DDSs) with high target specificity and less non-specific toxicity *in vivo*. Particularly, for ovarian cancer, DDSs will be useful to treat metastatic cancer cells in peritoneal cavity (Amreddy et al., 2018; van den Brand et al., 2018). Moreover, apoptosis-related lncRNAs may be potential diagnostic and prognostic biomarkers. Especially, *FEZF1-AS1* and *UCA1* are detected in serum and exosomes recovered from serum of ovarian cancer patients, respectively, which suggested their potential as liquid biopsy markers for ovarian cancer.

Apoptosis-related lncRNAs have basically been studied using conventional ovarian cancer cell lines, and the functions of some lncRNAs have been examined by *in vitro* assays alone. For clinical application, it is required to elucidate the lncRNA functions *in vivo*. Moreover, previous studies have indicated some discrepancies between ovarian cancer cell lines and the original tumor clinical tissues in terms of genomic and histological features and gene expression profiles (Domcke et al., 2013; Beaufort et al., 2014). Thus, lncRNA studies using ovarian tumor specimens or other ovarian cancer models are strongly demanded. Three-dimensional cultures of patient-derived cancer cells (PDCs) and cancer models established by transplanting tumor specimens into host mice (patient-derived xenograft [PDX] models) retain the properties of original tumors and have attracted attention as promising models for cancer research and drug screening (Ishiguro et al., 2016; Maru and Hippo, 2019; Namekawa et al., 2019; Shiba et al., 2019). Further studies using PDC and PDX models would advance the application of apoptosis-related lncRNAs to ovarian cancer diagnosis, prognosis, and therapies.

## Author Contributions

All authors contributed to the conception and provided the data and design. TT contributed to manuscript writing. KI, KH-I, and SI contributed to the conception and final approval of the manuscript.

## Conflict of Interest

The authors declare that the research was conducted in the absence of any commercial or financial relationships that could be construed as a potential conflict of interest.
